# Identification of Passion Fruit Oil Adulteration by Chemometric Analysis of FTIR Spectra

**DOI:** 10.3390/molecules24183219

**Published:** 2019-09-04

**Authors:** Johannes Kiefer, Anja I. Lampe, Stefano F. Nicoli, Massimo Lucarini, Alessandra Durazzo

**Affiliations:** 1 Technische Thermodynamik, Universität Bremen, Badgasteiner Str. 1, 28359 Bremen, Germany; 2 MAPEX Center for Materials and Processes, Universität Bremen, 28359 Bremen, Germany; 3 School of Engineering, University of Aberdeen, Aberdeen AB24 3UE, UK; 4 Erlangen Graduate School in Advanced Optical Technologies (SAOT), Friedrich-Alexander-Universität Erlangen-Nürnberg, 91052 Erlangen, Germany; 5 Consiglio per la ricerca in agricoltura e l’analisi dell’economia agraria-Centro di ricerca CREA-Alimenti e Nutrizione, Via Ardeatina 546, 00178 Roma, Italy (S.F.N.) (M.L.) (A.D.)

**Keywords:** passion fruit oil, maracuja oil, infrared spectroscopy, chemometrics, principal component analysis

## Abstract

Passion fruit oil is a high-value product with applications in the food and cosmetic sectors. It is frequently diluted with sunflower oil. Sunflower oil is also a potential adulterant as its addition does not notably alter the appearance of the passion fruit oil. In this paper, we show that this is also true for the FTIR spectrum. However, the chemometric analysis of the data changes this situation. Principal component analysis (PCA) enables not only the straightforward discrimination of pure passion fruit oil and adulterated samples but also the unambiguous classification of passion fruit oil products from five different manufacturers. Even small amounts—significantly below 1%—of the adulterant can be detected. Furthermore, partial least-squares regression (PLSR) facilitates the quantification of the amount of sunflower oil added to the passion fruit oil. The results demonstrate that the combination of FTIR spectroscopy and chemometric data analysis is a very powerful tool to analyze passion fruit oil.

## 1. Introduction

Passion fruit (*Passiflora edulis*) aka Maracuja is a popular fruit that is mainly used to make juices and desserts. Beyond this, however, the seeds are also known as a rich source of lipids, proteins, minerals, fiber, and a number of bioactive compounds such as sterols, tocols, and carotenoids [1]. Consequently, the seeds have gained increasing interest as a feedstock of oil for cosmetic and nutritional purposes. The chemical composition of the resulting passion fruit oils has been studied a number of times [2,3,4]. Concerning the fatty acid composition, the main constituents are linoleic, oleic, and palmitic acid [5,6,7]. The high content of polyunsaturated fatty acids and bioactive compounds has sparked an interest in the potential health-promoting properties of passion fruit oil. For example, there are studies on the antioxidant, antitumor, and antibacterial activities of passion fruit seed oil and its constituents [8,9,10].

Owing to the beneficial properties and the limited availability, passion fruit oil is an expensive product with prices between 50 and 200 USD per liter. As a consequence, it is often blended with other types of oils. For example, in cosmetics, it may be mixed with pracaxi oil while in other products [11], like food, blends with olive and sunflower oil are common. Such blending may also be done to dilute the expensive oil in order to make a larger profit. Pracaxi oil itself is a high-value product [12,13] and, hence, it is unlikely to be used to dilute or adulterate passion fruit oil. On the other hand, olive oil and sunflower oil can be produced significantly cheaper and therefore may be seen as potential adulterants. Olive oil has a rather characteristic odor [14], which makes it easy to detect by the human nose. In contrast, sunflower oil smells relatively neutral and thus it is most likely used to dilute passion fruit oil. Consequently, there is a need for a suitable analytical method that is capable of (1) distinguishing between sunflower and passion fruit oil and (2) determining the composition of their blends. Spectroscopic methods such as Fourier-transform infrared (FTIR) and UV/vis absorption spectroscopy are, generally, possible options for rapid authentication and detection of adulteration in food [15]; in particular, they are frequently used for oil analysis in the food and cosmetics sectors [16,17,18].

Studies on the application of advanced analytical methods to passion fruit oil are scarce. Ferreira et al. [19] employed Raman spectroscopy to investigate a number of Amazonian oils including passion fruit oil. They demonstrated the identification of typical carotenoid bands and succeeded in distinguishing between the different oil types. Raman spectroscopy in the near-infrared spectral range was therefore concluded to be a suitable tool for quality control of oils. Another study on quality control of Amazonian oils was performed using matrix-assisted laser desorption/ionization time-of-flight (MALDI-TOF) mass spectrometry utilizing the signatures of triacylglycerol [20]. De Vasconcelos Vieira Lopes et al. [21] applied ^1^H-NMR to passion fruit oil aiming at determining the degree of saturation. Furthermore, they demonstrated that the effects of desaturation of the fatty acids can be analyzed using ^1^H-NMR.

In comparison with the above methods, FTIR spectroscopy is more straightforward and robust, and it can easily be applied to virtually any kind of sample regardless of the shape or physical state [22,23]. Therefore, it is well suited for analyzing food products such as oils, but it has not been applied to passion fruit seed oils to date. Generally, infrared spectroscopy is an absorption method. The experimentally simplest and most robust approach to FTIR spectroscopy is attenuated total reflection (ATR) spectroscopy. In an ATR-FTIR experiment, the infrared radiation is propagating in a transmissive high-refractive-index material, often germanium, zinc selenide, or a diamond crystal. The sample has a lower refractive index and is in contact with the surface of the crystal. The radiation undergoes total internal reflection at this surface. The evanescent field interacts with the sample and the reflected beam is attenuated and carries the spectroscopic information. Details can be found in the literature [24,25]. One of the biggest advantages of this approach is that it requires virtually no sample preparation. Even opaque samples can be analyzed. The spectra obtained can be evaluated in many ways. In some cases, straightforward univariate methods such as the application of the Lambert–Beer law may be sufficient. If more complicated systems are studied, multivariate tools are required. For example, when the aim of the analysis is to classify an oil or to identify adulteration, chemometric classification tools are best suited. The most prominent and popular method for such data analysis is principal component analysis (PCA) [26,27,28,29]. PCA is a purely mathematical tool that determines the eigenvectors and eigenvalues of the data set. It returns the spectral signatures that contribute to the variance of the spectra in descending order. The first principal component represents the features that are characteristic of the largest variance and so on. Plotting the scores of the principal components (PCs) against each other shows whether or not the individual spectra in the data set group together to form certain clusters. Consequently, if different oils have sufficiently different spectra, they will be separated from each other in such a plot. This is not only true for oil FTIR spectra but for vibrational spectra from any food product [30,31,32]. On the other hand, in order to derive quantitative information from the data set, regression algorithms such as partial least-squares regression (PLSR) and support vector machine (SVM) regression are usually most appropriate [33,34,35,36].

In the present work, ATR-FTIR spectroscopy is applied to passion fruit oil and its blends with sunflower oil. The objectives of this study are (a) to demonstrate that FTIR spectra can be used authenticate and classify passion fruit oil and (b) to detect and quantify adulterants. For this purpose, five different passion fruit oil products have been studied. The experimental data have been evaluated using PCA and PLSR in order to test whether or not the different products can be discriminated and potential adulteration with sunflower oil can be detected and quantified.

## 2. Results and Discussion

In the following, we first present the fatty acid profiles of the PF seed oils as a reference for the analysis of the spectra, which are evaluated thereafter. We start with a brief interpretation of the FTIR spectrum of passion fruit oil. Then, a qualitative analysis is carried out aiming at a classification of the samples. Eventually, the quantitative assessment of the oil blends is the goal.

### 2.1. Fatty Acid Profiles

The results of the fatty acid analysis are summarized in Table 1 together with the concentration ranges typical for passion fruit oils. If an oil is slightly out of these ranges, it does not necessarily mean that it is counterfeit, but the overall picture allows drawing a conclusion. Regarding individual fatty acids, we limit ourselves to selected characteristic components: lauric acid (C14:0), palmitic acid (C16:0), stearic acid (C18:0), oleic acid (C18:1), linoleic acid (C18:2), and linolenic acid (C18:3).

The values of lauric and stearic acid are all within the typical ranges. Regarding palmitic acid, PF1, PF3, and PF5 are slightly above the typical range, but not far off. Looking at oleic acid, PF1, PF2, and PF4 are moderately above the typical range. On the other hand, these two products have slightly higher linoleic acid contents than the other oils. PF2 and PF4 are slightly below the typical linoleic acid range, while the value of PF1 is significantly lower. Considering linolenic acid, PF2–5 are within or slightly above (see PF2) the typical margin, but PF1 has a more than 12-fold value here. The latter in particular can hardly be explained by regional differences in the raw product and therefore PF1 is probably not a pure passion fruit oil. So the orange color (see Section 3) may not only be a result of a small amount of colorant like carotene. In order to shed further light at this point, Table 2 summarizes the fatty acid profiles of three potential adulterants for a comparison: sunflower oil, rapeseed oil, and olive oil. The high linolenic acid content of the PF1 product suggests that it was adulterated with rapeseed oil.

### 2.2. The Spectrum of Passion Fruit Oil

**Figure 1:**
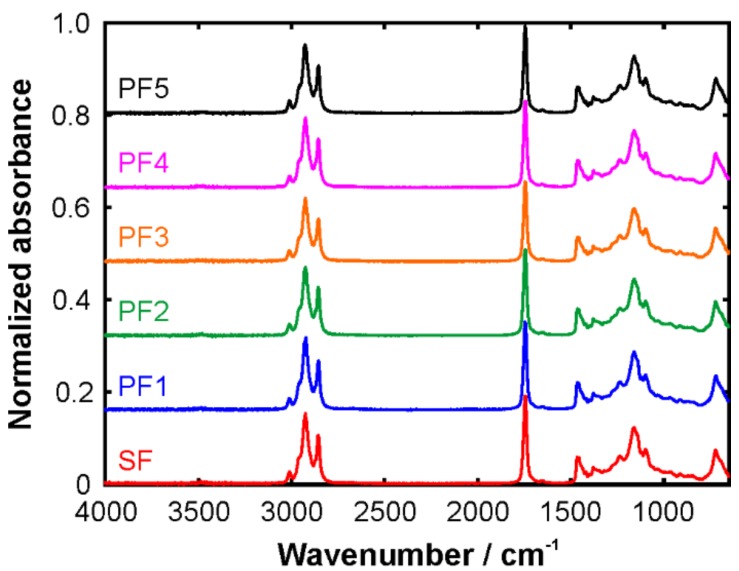
FTIR spectra of the different oil samples. PF indicates the passion fruit oils and SF marks the sunflower oil.

We proceed with the analysis of the FTIR spectra. Figure 1 shows the spectra of the five passion fruit oil samples in comparison with the sunflower oil; the corresponding example raw data are provided in the Supplementary Materials. At first glance, all six spectra look very similar. The very weak signature around 3500 cm^−1^ can have two origins: (1) OH stretching vibrations from the small amounts of water and (2) the overtone vibration of the strong C=O stretching mode in the ester groups. Fitting a Gaussian profile to this weak band yields a center wavenumber of 3481 (±5) cm^−1^. Between 2800 and 3100 cm^−1^, the CH stretching bands can be observed. Owing to the chemical complexity of the triglyceride molecules in the oils, this band constitutes a multitude of individual sub-peaks originating from different CH groups and their symmetric and anti-symmetric vibrational modes. The strong peak at ~1740 cm^−1^ is characteristic of the C=O double bonds in the ester groups. Depending on the sample, it is located between 1740 and 1746 cm^−1^ as was determined by fitting a sum of Gaussian profiles to the entire band from 1600 to 1850 cm^−1^. Its two-fold wavenumber almost perfectly matches the position of the weak signal at 3481 cm^−1^ lending further support for the assignment to an overtone vibration. The fingerprint region below 1700 cm^−1^ is very rich in content with overlapping peaks from stretching, rocking, wagging, and scissoring modes. In this region of the spectrum, it is difficult to make unambiguous assignments. With the naked eye, it is rather difficult to find notable differences in the passion fruit and sunflower oil spectra from Figure 1.

### 2.3. Product Discrimination and Detection of Adulteration

In order to discriminate between the different oil samples, a two-step evaluation using PCA was performed. In the first step, PCA was applied to the dataset consisting of the spectra from all six pure oils, PF1-5 and SF.

**Figure 2:**
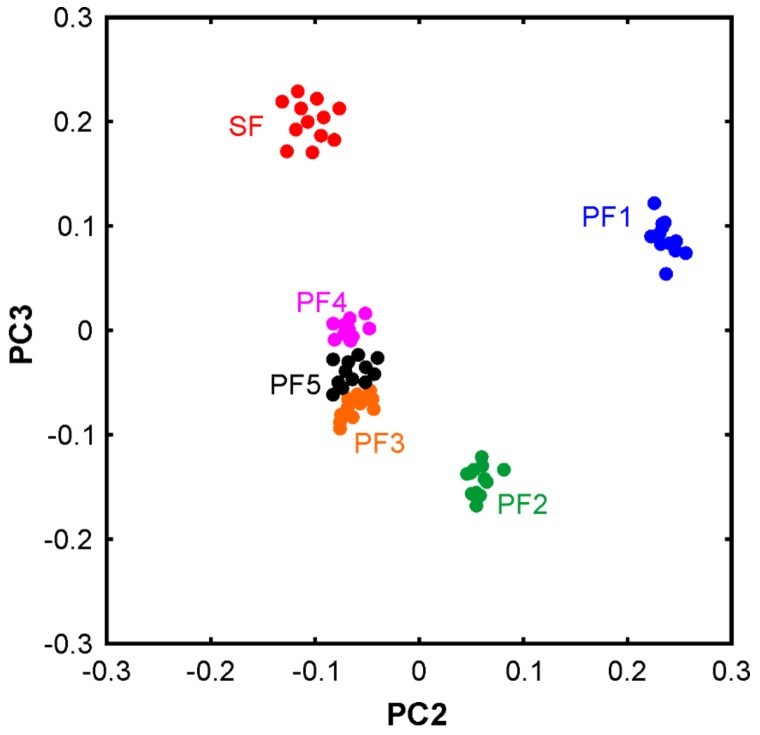
Principal component analysis of the six pure oil samples. Variance covered: PC1 (99.9208%), PC2 (0.0270%), and PC3 (0.0065).

**Figure A1:**
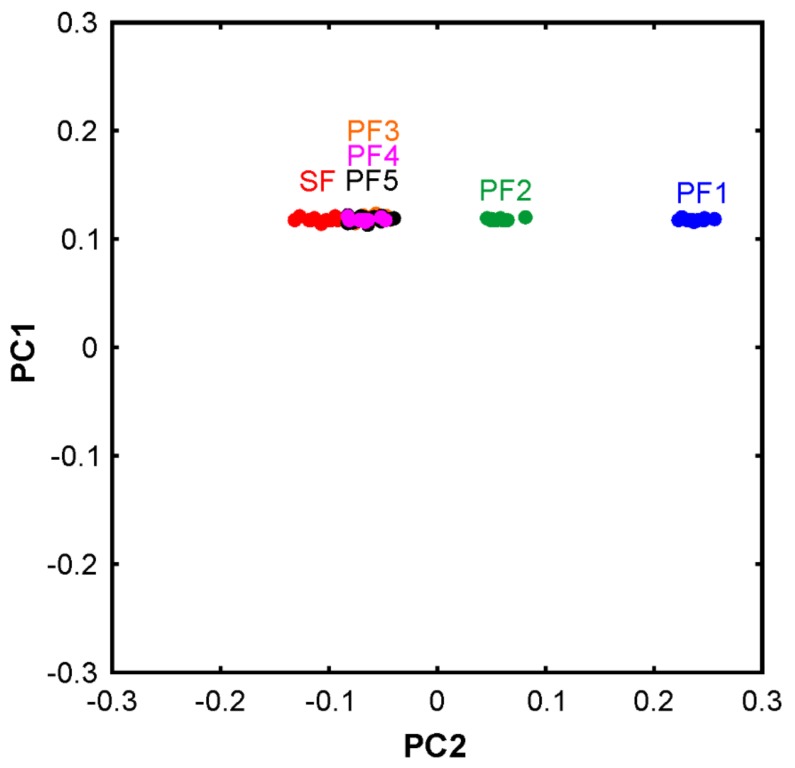
Score plot of the principal component analysis of the six pure oil samples: PC1 vs. PC2. As the data were not mean-centered, the spectra cannot be classified along PC1. In other words, all samples exhibit essentially the same PC1 value.

Figure 2 shows the 2D plot of the scores of the third vs. second principal component, PC3 and PC2, respectively. As the data were not mean-centered, PC1 basically describes the mean spectrum and therefore is not included. In other words, the separation of the different samples along PC1 is essentially zero as shown in Figure A1. For completeness we note that mean centering would result in the spectral variability to be mainly represented in the first two PCs, but the overall outcome of the classification via PCA would be the same. Despite the negligible visible differences, the unsupervised PCA yields a clear discrimination between the sunflower oil and passion fruit oil samples. Furthermore, PF1 and PF2 are clearly separated from the other three passion fruit oils. This is remarkable as PF1 and PF2 are the two samples that have rather high contents of C18:3. As we have discussed above, in PF1 the content is so high that this sample is probably not a pure passion fruit oil. PF3 and PF5 are grouping closely together as it can be expected from their similar fatty acid profiles. These are the two samples that are sold as food rather than cosmetic products. Different processing or small amounts of additives in the cosmetic and food products may explain the differences in the fatty acid profiles and in the location in the score plot. For instance, the oil used for food purposes may have been extracted with green technologies and treated more gently. Detailed information about the production was not available, unfortunately. Nevertheless, these results point towards new applications of FTIR (or vibrational spectroscopy in general) for product authentication if it is possible to draw conclusions about the processing. We already speculated about that in a recent book chapter [40], but the present results and a previous study on Scotch Whiskies [30], in which Raman spectra revealed information about the processing, highlight that these directions are very promising indeed.

The PF3, 4, and 5 samples appear close to each other with some minor overlap, so in principle, they can be discriminated already in this first step. The separation of SF, PF1, and the remaining group of passion fruit oils are very clear so that it is likely to find small but distinct spectral differences in the raw data. Therefore, all spectra were plotted with a color code in a sense that the following groups can be distinguished: (i) all SF spectra, all PF1 spectra, all PF3 spectra, and all PF spectra. The PF3 spectra are selected here as the data revealed one very small but systematic difference.

**Figure 3:**
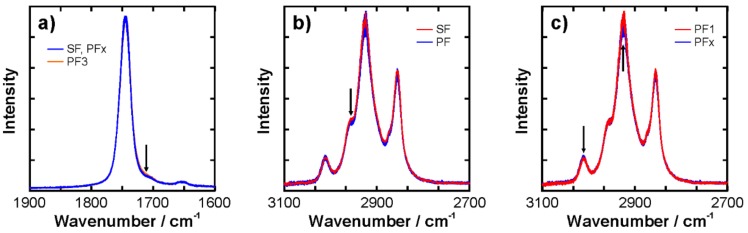
Color-coded raw spectra in selected spectral ranges: (**a**) C=O stretching region and (**b**,**c**) CH stretching region.

Selected ranges of the resulting plots are displayed in Figure 3. Panel (a) shows the enlarged C=O stretching band, which can be attributed to the ester groups. The main band is located at 1740 cm^−1^ and all the spectra overlap without a notable separation of the individual groups marked by a different color. However, the shoulder band at 1710 cm^−1^ allows the ability to distinguish between the PF3 spectra and all other samples. A similar sideband was recently reported for blends of commercial diesel and butanol [41], where it was attributed to biodiesel ester groups interacting with the hydroxyl groups of the alcohol. Such a hydrogen bonding interaction can lead to a peak shift like the one observed [42]. However, this would indicate the presence of molecules with OH groups in the PF3 oil sample. As the CH stretching region exhibits very similar bands for all passion fruits oils (see Figure 3b), it is unlikely that alcohol groups are the origin of the observed band. The presence of water would be another explanation. As stated in Section 3, the water content of all samples was below 1000 ppm. Revisiting the data in detail, however, shows that all the samples had a water content between 200 and 350 ppm, but the PF3 oil contained ~780 ppm. Due to the substantial difference in molecular weight between the water and oil molecules, there is a measurable number of ester groups interacting with water. Therefore, we conclude that the observed shoulder band results from the ester groups that are hydrogen-bonded to water molecules.

The CH stretching region in Figure 3b, on the other hand, reveals small but systematic differences between the passion fruit and sunflower oil spectra. This can be attributed, e.g., to differences in the fatty acids content. For example, passion fruit seed oil has larger amounts of unsaturated fatty acids than sunflower oil. Analyzing the PF samples in this manner yields the plot in Figure 3c. The spectra of PF1 are now in red and show subtle but notable differences to the other PF oils: the peak at 3013 cm^−1^ is tentatively weaker in PF1 and the peak at 2927 cm^−1^ is tentatively slightly stronger than in the other PF oils spectra. Those peaks can be assigned to the =C-H stretching and asymmetric CH_2_ stretching modes [43]. In other words, the spectra indicate that PF1 contains a lower number of unsaturated C=C bonds. In order to compare this hypothesis with the fatty acid profiles discussed above, a rough calculation was done: for each fatty acid the number of double bonds was multiplied with the percentage in Table 1 and the results were summed up to yield a single number for each sample. For example, for PF1 we calculate: 0.08 × 0 + 12.90 × 0 + 3.50 × 0 + 24.79 × 1 + 52.17 × 2 + 6.56 × 3 = 148.81. Given the differences in molecular mass and density of the different constituents, the resulting number is not an accurate measure of the number of double bonds in the oils, but it provides a rough idea. From this estimation, we obtain 148.8 for PF1, 154.3 for PF2, 153.8 for PF3, 153.5 for PF4, and 153.9 for PF5. The number for PF2–5 are remarkably close to each other, while PF1 is significantly lower. These numbers are also reflected in the fatty acid sums given in Table 1. While the overall amount of unsaturated fatty acids is rather similar in all products, the polyunsaturated fatty acids distinguish PF1 from the others. This agrees well with the conclusion drawn from the spectra and corroborates our hypothesis that PF1 is not a pure passion fruit oil.

**Figure 4:**
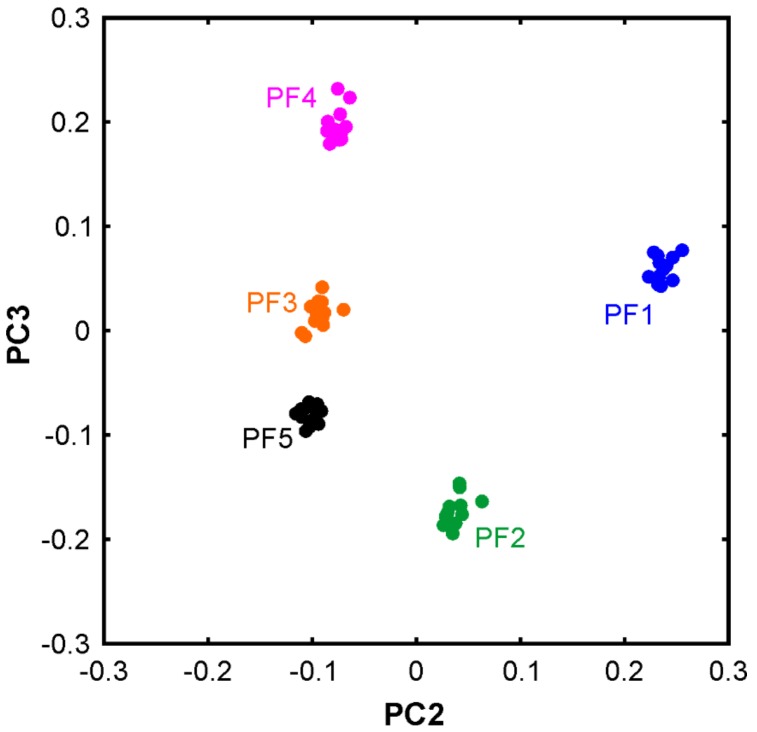
Principal component analysis of the five passion fruit oil samples. Variance covered: PC1 (99.9210%), PC2 (0.0285%), and PC3 (0.0064).

In the second step, the PCA was applied to a reduced dataset from which the sunflower oil spectra were removed. Figure 4 shows the corresponding 2D plot of the scores of PC3 against PC2. All five passion fruit oils can be unambiguously distinguished. The variance covered by the first three PCs is 99.9210%, 0.0285%, and 0.0064% in ascending order. Although the variance covered by PC2 and PC3 is rather small, the corresponding plots of the loading vs. wavenumber exhibit a sufficiently high signal-to-noise ratio (>15). This indicates that all three PCs are meaningful.

**Figure 5:**
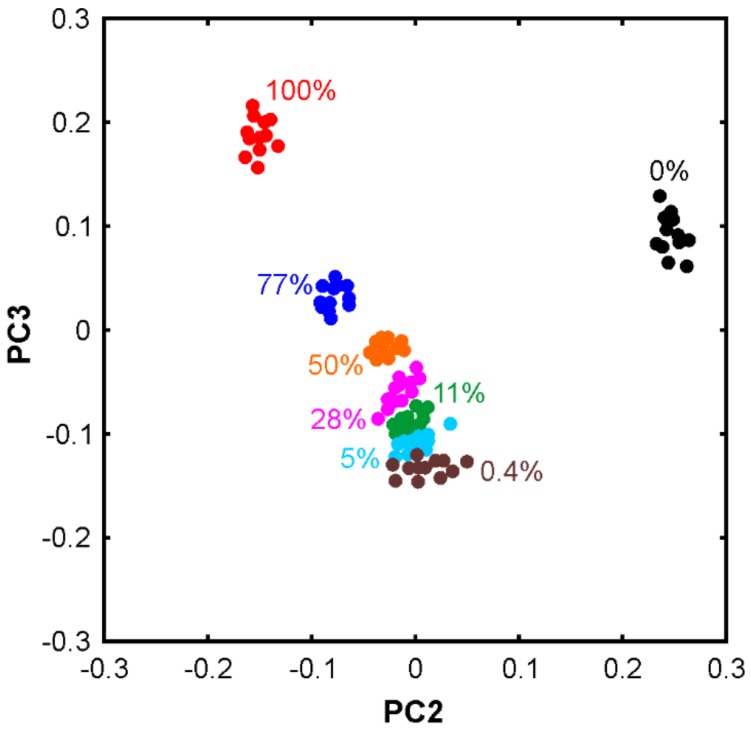
Principal component analysis of the passion fruit oil blends with sunflower oil. The percentages indicate the mass fraction of sunflower oil. Variance covered: PC1 (99.9326%), PC2 (0.0151%), and PC3 (0.0052).

In order to assess whether or not PCA can also be utilized for discriminating between a pure passion fruit oil and adulterated samples, the method has been applied to the spectra of the blends. Figure 5 displays the scores plot (PC3 vs. PC2) of the blends of the sunflower oil and PF3. The percentages indicate the mass fraction of sunflower oil, i.e., the adulterant.

**Figure 6:**
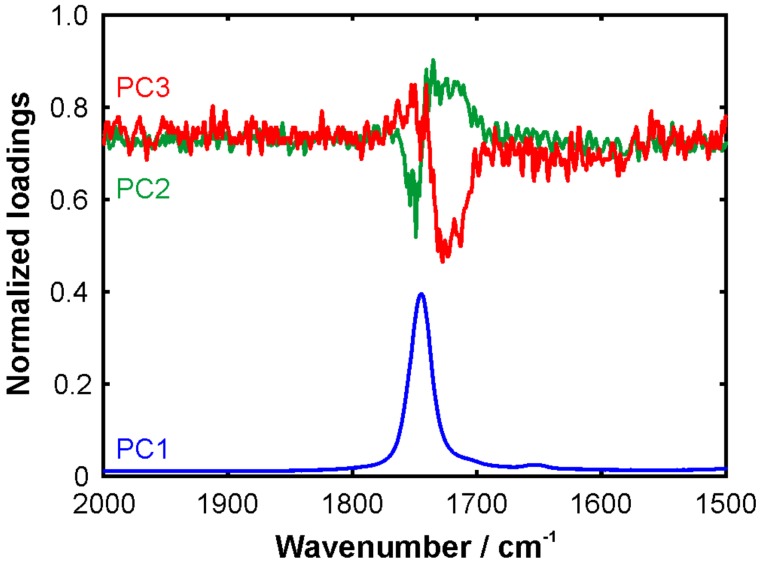
Loadings plot from the PCA of the passion fruit oil blends with sunflower oil showing the intensity normalized PCs 1–3 in the spectral range of the carbonyl stretching mode.

The results are very promising from a food analysis point of view. First of all, the sunflower oil, passion fruit oil, and their blends can be distinguished clearly. Secondly, the blends follow an explicit trend indicating that quantification is possible. It is rather surprising that the sample with only 0.4% sunflower oil is still clearly separated from the pure passion fruit oil. This suggests that the detection limit of sunflower oil in passion fruit oil is estimated to be at the 100 ppm level. In other words, adulterated passion fruit oil can easily be identified using the proposed combination of FTIR spectroscopy and PCA. Another surprising point is that the blends are not located between the pure oils. First of all, we note that the data points lie on such a line between the pure oils when plotting PC2 or PC3 against PC1. The deviation in the PC3 vs. PC2 plot could be interpreted in such a way that the molecular interactions between the SF and PF oils components result in a characteristic spectral signature that is picked up by the PCA algorithm. The loadings plot supports this hypothesis in particular in the spectral region of the C=O stretching band, see Figure 6. PC2 and PC3 show mirrored S-shaped signatures indicating intermolecular interactions involving the carbonyl groups of the ester moieties. However, a more detailed analysis of these effects is beyond the scope of the present work.

### 2.4. Quantitative Analysis of Oil Mixtures

**Figure 7:**
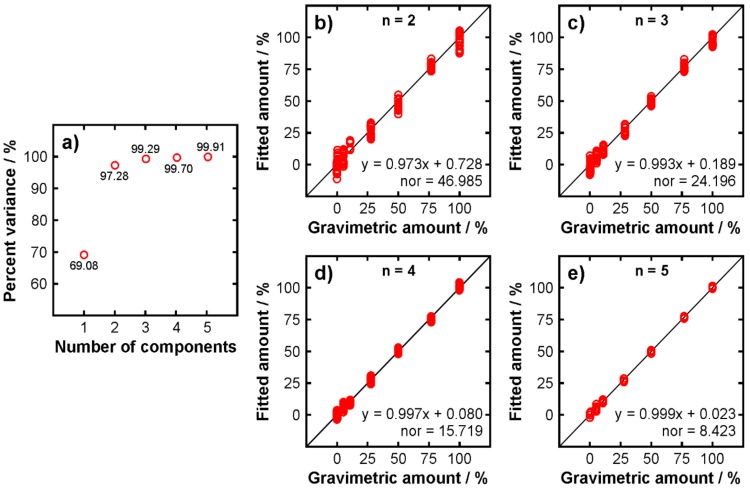
Partial least-squares regression (PLSR) results of the blends. (**a**) Cumulative variance explained by the first 5 PCs; (**b**–**e**) fitted results versus gravimetrically set amount of sunflower oil in the blends: evaluation utilizing different number of components (n). The equations are linear functions fitted to the experimental data in order to assess the quality of the evaluation. The norm of residuals (nor) is provided as a measure for the goodness of the corresponding fit.

In the final part of this study, PLSR was applied to the spectra of the blends in order to investigate its potential for quantifying the amount of adulterant. Using PLSR bears some danger as utilizing a large number of components will always yield nice-looking regression results. However, higher components typically comprise of noise predominantly and hence including them does not make sense. Figure 7a displays the accumulative variance of the dataset explained by the first five PLS components. It can be seen that the first three components are responsible for about 99.3% of the variance of the dataset. The fourth component adds another 0.4%, which may still be seen sufficiently significant, but the components beyond number 4 each add 0.2% and less.

A comparison was carried out by performing PLSR with different numbers of components from two to five. The results are plotted in Figure 7b–e, respectively, representing the prediction of the calibration samples that were used to build the calibration model. Already with two components (Figure 7b) a reasonable correlation is produced. The individual data points scatter moderately. The solid lines in the individual panels represent the y = x function, i.e., the predicted values from PLSR are identical with the gravimetric values set. The scatter of individual data point on the *y*-axis reduces with increasing number of components. In order to further quantify the results, the data points were fitted by a linear function for each case. The resulting equations, as well as the norms of residuals of the fitting, are displayed in the individual panels. Ideally, the slope of these functions should be equal to unity and the axis intercept should be zero. As can be seen, the slope and the intercept approach one and zero, respectively, when the number of components increases. However, as discussed above, employing *n* > 4 does not make sense as the higher components are not meaningful. Nevertheless, the results demonstrate that the amount of adulterant can be quantified with reasonable accuracy and precision. Although it should be noted that the measurement uncertainty increases with decreasing concentration as indicated by the scatter of the data points.

**Figure 8:**
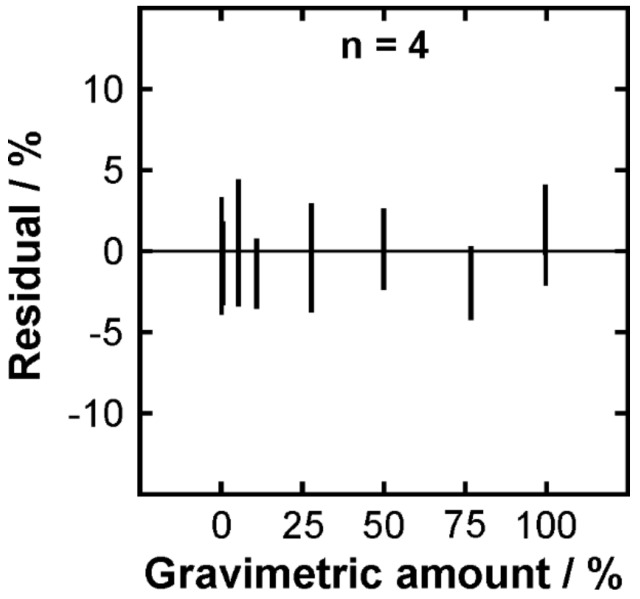
Residual ranges of the leave-one-out cross-validation (LOOCV) for the PLSR evaluation employing four components.

In order to test the robustness of the PLSR approach, a leave-one-out cross-validation (LOOCV) was performed using the *n* = 4 case as an example. Figure 8 illustrates the residual ranges for the individual gravimetric amounts. It can be seen that there are no systematic deviations indicating that the developed model is suitable. The deviation ranges cover a few percent around zero suggesting a good reproducibility and robustness of the method.

## 3. Materials and Methods

Passion fruit oil products were purchased from five different producers and used as received. In the paper they are termed PF1-5: PF1 is a product from Brazil; PF2 is a product from India; PF3 is a product from Germany; PF4 is a product from Poland; PF5 is a product from Germany. According to the producers, they all consist of 100% passion fruit oil. PF2-5 appear as shale yellowish oils while PF1 has an orange color, which suggests at first glance that at least a small amount of colorant (for example, beta-carotene dye) was added. PF1, 2, and 4 are sold as cosmetic products, while PF3 and 5 are sold as edible oils for the preparation of food. For all samples, the water content was measured to be below 1000 ppm by Karl Fischer titration. In addition, a sunflower oil (brand “gut und günstig”) was purchased from a local supermarket (Edeka, Germany) for comparison. This particular sunflower oil was identified as a good representative amongst a selection of seven pure sunflower oils. It is a standard sunflower oil (not a high oleic product). All six oils were studied in their pure form without further purification or pre-treatment. Furthermore, blends of passion fruit and sunflower oil were prepared gravimetrically using a microbalance. For each blend, a total of about 3 g was mixed in PTFE-sealed Duran type culture tubes (Duran Group, Mainz, Germany). The sunflower oil mass percentages of the PF3 blends were 77, 50, 28, 11, 5, and 0.4. For completeness, we note that we have also studied sunflower oil blends with the other PF (PF2, 4, and 5) samples, but the results were essentially the same.

The FTIR spectra of the oils and blends were recorded on an Agilent Cary 630 instrument equipped with a ZnSe unit for attenuated total reflection operation (5 bounces at 45°). Prior to a measurement, the ATR unit was carefully cleaned with ethanol and acetone. Then, one droplet of the oil or blend was placed on the ATR crystal and covered with a glass lid in order to avoid contamination with ambient moisture. For each sample, 12 spectra were recorded, and for each spectrum, 16 scans were averaged in order to obtain an appropriate signal to noise ratio. The spectral range was 650–4000 cm^−1^ and the nominal resolution was 2 cm^−1^. The spectra were evaluated using principal component analysis (PCA) and partial least-squares regression (PLRS). The spectra were not pretreated before the chemometric analysis; neither a baseline correction nor a smoothing nor any other form of data manipulation was applied. The evaluation algorithms were implemented in Matlab.

In order to have confidence in the samples under study and to aid the spectroscopic analysis, the fatty acid profiles of the five passion fruit oils were analyzed using a gas chromatograph (Agilent 7890A), equipped with both FID and MS (Agilent 5975C) detectors. The separation of the fatty acids was accomplished on a Mega-wax column (30 m × 0.32 mm i.d., 0.25 µm film thickness). The GC system allows to acquire and record in the same injection both the FID and MS signals, for quantitative and qualitative determinations, respectively. Identification was also carried out by comparing the retention time of the detected compounds in the sample with those from a standard FAME mix (Supelco TM 37 component FAME mix C4-C24; Sigma-Aldrich, St Louis, MO, USA). Quantification was performed calculating the internal percentage distribution of FAME.

## 4. Conclusions

In this article, we have demonstrated that FTIR spectroscopy in combination with multivariate data evaluation in terms of principal component analysis (PCA) and partial least-squares regression (PLSR) is a powerful tool for the analysis of passion fruit oil. Despite very small spectral differences, the chemometric methods allow an unambiguous discrimination between passion fruit oil and sunflower oil, which is a potential adulterant. Furthermore, even passion fruit oil samples from different producers and for different fields of application (food vs. cosmetic) can be distinguished. The spectra revealed that one product tested was likely not a pure passion fruit oil. This was also concluded from the fatty acid profiles determined by gas chromatography. In the second part of the study, blends of passion fruit oil and sunflower oil were analyzed in order to evaluate whether or not the analytical method is capable of detecting adulteration and quantifying the amount of the adulterant. The results evidence that even small amounts of sunflower oil, i.e., significantly below one percent, can be detected. In particular, the PCA turned out to be highly effective regarding the classification of adulterated samples. The quantification of the sunflower oil content was possible using PLSR. Sufficiently accurate results were obtained employing three or four components, but we note that two components already provided a reasonable correlation.

## Figures and Tables

**Table 1:** Fatty acid profiles of the passion fruit oil products. The numbers are mean values of multiple measurements and the standard deviations are given in parentheses. The numbers are expressed as percentages of the total fatty acid content. The typical ranges are given for comparison; these numbers were derived from the literature [5,37,38].

Fatty Acid	PF1	PF2	PF3	PF4	PF5	Typical Range
C14:0	0.08 (0.07)	0.05 (0.05)	0.07 (0.07)	0.00 (0.00)	0.07 (0.06)	<0.2%
C16:0	12.90 (0.50)	8.39 (0.55)	12.36 (0.88)	8.06 (0.49)	12.50 (0.21)	8–11%
C18:0	3.50 (0.06)	2.74 (0.48)	2.49 (0.28)	2.55 (0.57)	2.62 (0.05)	1–4%
C18:1	24.79 (0.56)	24.44 (1.54)	16.57 (0.87)	25.46 (1.36)	16.09 (0.41)	13–17%
C18:2	52.17 (0.54)	63.26 (2.25)	68.30 (0.57)	63.71 (2.38)	68.30 (0.54)	67–74%
C18:3	6.56 (0.88)	1.12 (0.24)	0.20 (0.19)	0.22 (0.03)	0.41 (0.05)	<0.5%
ΣUFA ^1^	83.52 (1.98)	88.82 (4.03)	85.07 (1.36)	89.39 (3.77)	84.88 (1.00)	
ΣMUFA ^2^	24.79 (0.56)	24.44 (1.54)	16.57 (0.87)	25.46 (1.36)	16.09 (0.41)	
ΣPUFA ^3^	58.73 (1.42)	64.38 (2.49)	68.50 (0.76)	63.93 (2.41)	68.71 (0.59)	

^1^ unsaturated fatty acids; ^2^ monounsaturated fatty acids; ^3^ polyunsaturated fatty acids.

**Table 2:** Fatty acid profiles of potential adulterants. The numbers are from the Codex Alimentarius 2001 of the Food and Agricultural Organization of the United Nations World Health Organization [39].

Fatty Acid	Sunflower	Rapeseed	Olive
C14:0	<0.2%	<0.2%	<0.1%
C16:0	5.0–7.6%	1.5–6.0%	7.5–20.0%
C18:0	2.7–6.5%	0.5–3.1%	0.5–5.0%
C18:1	14.0–39.4%	8–60%	55–83%
C18:2	48.8–74.0%	11–23%	3.5–21.0%
C18:3	<0.3%	5–13%	<1.5%

## Supplementary Materials

The following are available online, Table S1: raw data in csv format (columns: wavenumber in cm^−1^, absorbance values for SF, PF1-5).
